# Commentary on “The recent two decades of traumatic brain injury: a bibliometric analysis and systematic review”

**DOI:** 10.1097/MS9.0000000000002811

**Published:** 2024-12-19

**Authors:** Zengkun Fang, Pengke Pan

**Affiliations:** Department of Outpatient, Jiangbin Hospital of Guangxi Zhuang Autonomous Region, Nanning, People’s Republic of China

*Dear Editor,*

We express our interest in the recent review article titled “The recent two decades of traumatic brain injury: a bibliometric analysis and systematic review” published in the *International Journal of Surgery*^[1]^. We appreciate Ziyin Ye and his team for their innovative research efforts.

The authors selected 41 545 papers related to traumatic brain injury (TBI) from the Web of Science Core Collection database and analyzed publications in the TBI field over the past two decades to uncover research trends, hotspots, and frontiers^[1]^.

TBI represents a significant public health challenge, resulting in personal health losses and disabilities. It imposes burdens on healthcare systems and the economy through productivity losses and high healthcare costs^[2]^. Addressing the personal and societal burden of TBI is a pressing concern for medical professionals, evidenced by the increasing research efforts in TBI, as highlighted by the annual publication analysis conducted by Ziyin Ye and his team^[1]^.

Collaborative research among different countries, institutions, and authors helps advance solutions to scientific questions. Ziyin Ye and his team displayed the collaboration networks in the TBI field within the supplementary materials of their article^[1]^. This helps analyze the current cooperation landscape and identify existing issues, promoting further collaboration. In their results on Co-authorship and cooperation analysis, they determined the size of nodes based on the number of documents. However, I believe it would be more effective to base node size on total link strength (TLS). The number of documents for key countries, institutions, and authors is already detailed in Tables 1, 2, and 3 of their article^[1]^. Therefore, it is unnecessary to repeat that in the co-authorship and cooperation analysis. In contrast, showcasing the values of TLS is crucial for assessing the overall cooperation levels of a country, institution, or author. This should be emphasized in the result of cooperation analysis.

In the keyword clustering analysis, Ziyin Ye and his team employed VOSviewer to generate colored clusters^[1]^, but VOSviewer does not provide cluster names definitely. Conversely, CiteSpace clusters can be named based on title words, keywords, subject categories, and title words of references^[3,4]^. Thus, CiteSpace is more commonly used for keyword clustering due to its clarity. Additionally, the article’s keyword timeline view in VOSviewer defaulted to a 5-year analysis from 2013 to 2017, which is insufficient considering Ziyin Ye and his team’s comprehensive 20-year literature search. Extending the time frame would better represent the occurrence sequence of keywords. This timeline view is also more intuitively presented using CiteSpace.

In conclusion, the findings from Ziyin Ye and his team undoubtedly provide researchers in the TBI field with valuable insights into research trends, hotspots, and innovations. However, there are opportunities for improvement in the presentation and analysis of specific data.

## Data Availability

The datasets produced in this study can be accessed upon reasonable request from the corresponding author.
